# Bacteriophage Distributions and Temporal Variability in the Ocean’s Interior

**DOI:** 10.1128/mBio.01903-17

**Published:** 2017-11-28

**Authors:** Elaine Luo, Frank O. Aylward, Daniel R. Mende, Edward F. DeLong

**Affiliations:** Daniel K. Inouye Center for Microbial Oceanography: Research and Education, University of Hawaii, Honolulu, Hawaii, USA; University of Georgia

**Keywords:** bacteriophages, bacterioplankton, marine microbiology, microbial ecology, microbial oceanography

## Abstract

Bacteriophages are numerically the most abundant DNA-containing entities in the oligotrophic ocean, yet how specific phage populations vary over time and space remains to be fully explored. Here, we conducted a metagenomic time-series survey of double-stranded DNA phages throughout the water column in the North Pacific Subtropical Gyre, encompassing 1.5 years from depths of 25 to 1,000 m. Viral gene sequences were identified in assembled metagenomic samples, yielding an estimated 172,385 different viral gene families. Viral marker gene distributions suggested that lysogeny was more prevalent at mesopelagic depths than in surface waters, consistent with prior prophage induction studies using mitomycin C. A total of 129 ALOHA viral genomes and genome fragments from 20 to 108 kbp were selected for further study, which represented the most abundant phages in the water column. Phage genotypes displayed discrete population structures. Most phages persisted throughout the time-series and displayed a strong depth structure that mirrored the stratified depth distributions of co-occurring bacterial taxa in the water column. Mesopelagic phages were distinct from surface water phages with respect to diversity, gene content, putative life histories, and temporal persistence, reflecting depth-dependent differences in host genomic architectures and phage reproductive strategies. The spatiotemporal distributions of the most abundant open-ocean bacteriophages that we report here provide new insight into viral temporal persistence, life history, and virus-host-environment interactions throughout the open-ocean water column.

## IMPORTANCE

The North Pacific Subtropical Gyre represents one of the largest biomes on the planet, where microbial communities are central mediators of ecosystem dynamics and global biogeochemical cycles. Critical members of these communities are the viruses of marine bacteria, which can alter microbial metabolism and significantly influence their survival and productivity. To better understand these viral assemblages, we conducted genomic analyses of planktonic viruses over a seasonal cycle to ocean depths of 1,000 m. We identified 172,385 different viral gene families and 129 unique virus genotypes in this open-ocean setting. The spatiotemporal distributions of the most abundant open-ocean viruses that we report here provide new insights into viral temporal variability, life history, and virus-host-environment interactions throughout the water column.

## INTRODUCTION

Viruses are abundant biotic entities that play critical roles in aquatic environments. Some of the most common among these viruses in the open ocean are double-stranded DNA (dsDNA) bacteriophages (phages) that infect many abundant and biogeochemically important groups of bacterioplankton, such as *Prochlorococcus*, *Synechococcus*, and numerous heterotrophic bacterial species in common genera such as *Roseobacter*, *Alteromonas*, *Pelagibacter*, and *Puniceispirillum* (1–6). Phages have been shown to kill hosts at rates of up to 20 to 40% of the total population per day, potentially strongly impacting bacterioplankton populations (7, 8). In addition, carbon flux through phage biomass is estimated at 145 gigatonnes per year, playing a substantial role in the global carbon cycle (9). Furthermore, phages can influence ocean biochemistry through microbial cell lysis leading to the production of dissolved organic matter (DOM) and via auxiliary metabolic genes (AMGs) that alter cellular carbon, sulfur, and nitrogen metabolism of their hosts during the phage replication cycle (10–14). Advancing fundamental knowledge of marine phages is therefore an important step toward developing a deeper understanding of marine ecosystem dynamics.

Phages have critical roles in the microbial ecology and biogeochemistry of the global ocean due to their tremendous abundance and diversity. While the genotypic diversity of marine phages has historically been difficult to ascertain, recent studies have provided new insights into viral genomic diversity in the oceans. Developments in high-throughput DNA sequencing have enabled the exploration of viral diversity in the environment at unprecedented scales (13–17). These frequent reports of large reservoirs of viral genetic diversity highlight the importance of further work of *in situ* characterization of novel marine phages using reference-independent metagenomic techniques that do not rely on a limited subset of cultivable hosts.

The majority of published marine viral metagenomic surveys to date have focused on cataloging the genomic diversity and geographic variability in surface water samples (14–16). The vertical and temporal distributions of environmental phage assemblages have received relatively less attention. To our knowledge, only two metagenomic studies have reported on phages recovered from deep-sea planktonic samples. The Pacific Ocean Virome data set included 12 samples from the deep Pacific revealing that aphotic zone viromes contained a unique set of AMGs that distinguish them from photic zone viromes (13), while the structure of 99 genomic fragments of bathypelagic phages has been reported from the Mediterranean Sea (17). These studies suggested that deep-ocean phages are largely novel and distinct from previously characterized surface phages, highlighting the need to explore the vast diversity of uncharacterized phages below the surface ocean. With respect to temporal variability, previous studies have focused on daily, weekly, or annual scales in surface waters (18–24). Additional metagenomic studies of depth profiles coupled with temporal variability should prove useful as well to provide further insight into the spatiotemporal dynamics of marine phage populations.

Elucidation of viral dynamics in space and time at well-defined study sites has potential to provide the environmental context for interpreting broader patterns and consequences of viral diversity. Here, we present a metagenomic depth profile time-series of phage genes, genomes, and genome fragments captured in cellular bacterioplankton fractions (>0.2 µm) from depths of 25 to 1,000 m over 1.5 years. We used two approaches to explore how phages vary through depth and time in the North Pacific Subtropical Gyre (NPSG), an oligotrophic habitat that is representative of the largest biome in the world (25). Using a genome-centric approach, we describe genomic fragments of abundant phage populations at Station ALOHA. Through implementation of a multistep reassembly workflow, we reconstructed viral population genomes to describe how the diversity, distribution, and genetic repertoire of phages vary through depth and time. In the second approach, we used a gene-centric methodology that leveraged a previous study of prokaryotic assemblages at Station ALOHA (26). Specifically, we used the previously reported nonredundant gene catalog constructed from Station ALOHA to analyze the vertical distribution of phage genes and examine how the diversity and functional repertoire of phages vary across depth profiles. We also used marker genes to explore how viral life-history strategies shift through the ocean’s water column. Our analyses characterize viral gene distributions, genotypes, and temporal dynamics across a range of depths and provide important insight into the genomic diversity and dynamics of viral assemblages in the open ocean.

## RESULTS AND DISCUSSION

Viral genotypic diversity was inferred from metagenomic data collected at Station ALOHA from 25 to 1,000 m over 1.5 years, using both genome-centric and gene-centric approaches (see Fig. S2 in the supplemental material). The genome-centric methodologies used generated 129 viral contigs between 20 and 108 kbp in length, representing genomes or large genomic fragments of some of the most abundant phages at Station ALOHA. These were used to characterize the distributions of dominant viral populations and provide genomic insights into the ecology of specific phage groupings. In addition, the gene-centric approach of all Station ALOHA contigs assembled (26) captured a wide range of viral diversity from 177,713 nonredundant viral genes, which facilitated broader quantitative analyses of phage gene distributions in space and time, providing insight into the relationships between viral life history, environmental, and host variability.

10.1128/mBio.01903-17.1 FIG S1  Our data set includes 83 samples from 7 depths and 12 time points from August 2010 to December 2011. Download FIG S1, PDF file, 0.5 MB. Copyright © 2017 Luo et al. 2017 Luo et al. This content is distributed under the terms of the Creative Commons Attribution 4.0 International license.

10.1128/mBio.01903-17.2 FIG S2  Bioinformatic workflow from sequencing to gene- and genome-centric approaches. Download FIG S2, PDF file, 0.1 MB. Copyright © 2017 Luo et al. 2017 Luo et al. This content is distributed under the terms of the Creative Commons Attribution 4.0 International license.

### Novel ALOHA viral genomes, genome fragments, and AMGs.

**FIG 1:**
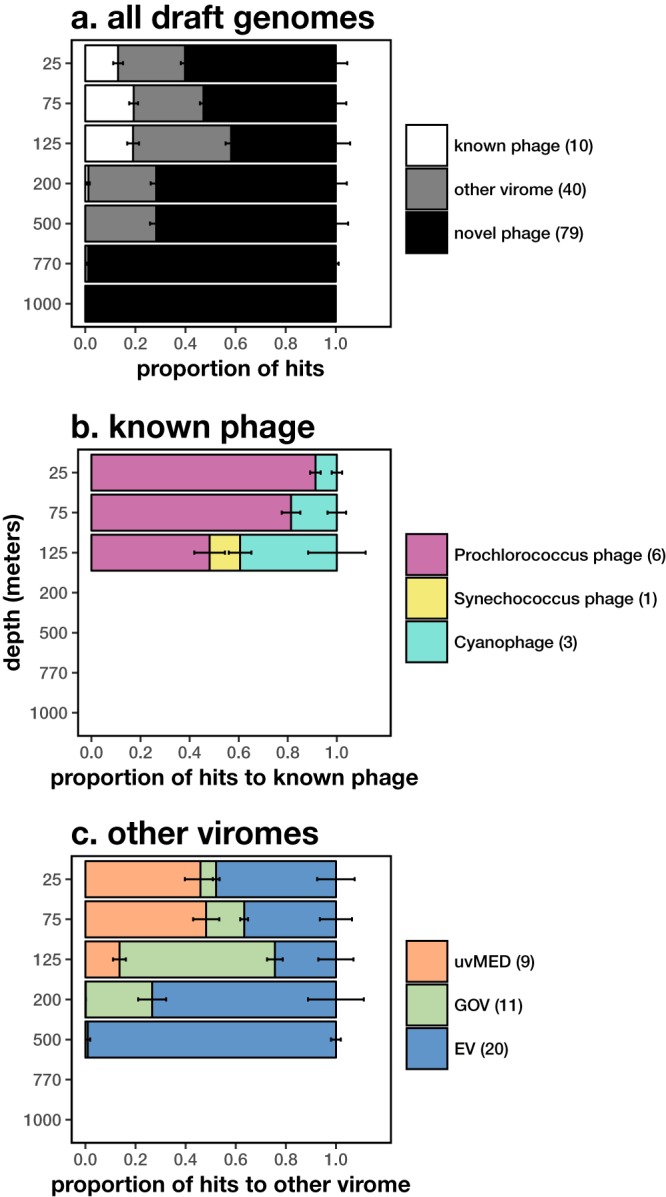
Depth profiles of known and novel phage ALOHA viral contigs assigned using four reference databases: known phages in RefSeq75 protein database and three previously sequenced viral metagenomes. Phages are identified with a cutoff of >50% genes with >60% average amino acid identity to a reference database genome. (a) Proportion of contigs with hits to known phages in RefSeq75, other viromes, and novel phages. (b) Subset of hits to RefSeq75. (c) Subset of hits to other viromes: uvMED from the Mediterranean (15), Global Ocean Viromes (GOV) from Tara Oceans (14), and Earth viromes (EV) from human, terrestrial, and marine environments (27). The number of contigs in each category is shown in the legend. Proportion is normalized by total nucleotides mapped to each contig. Error bars show standard errors, which are summed among groups in stacked bars.

Our conservative viral genome assembly strategy yielded many novel viral populations different from previously characterized viruses (Fig. 1). Seventy-nine out of 129 ALOHA viral contigs shared relatively little sequence homology to any known phages or previously sequenced viral metagenomes. Of the 50 Station ALOHA viral contigs having some database homologues, 10 were related to known phage genomes in RefSeq75 (all cyanophages, Table S2), while 40 shared homology to phages in one of three previously sequenced viral metagenomes (14, 15, 27). These data suggested that at least some of the most abundant phage groups that we found were widespread across ocean basins.

10.1128/mBio.01903-17.6 TABLE S1  Assembly statistics for the initial individual assemblies for each sample, number of contigs >3 kb in length used for VirSorter runs, and number of VirSorter identified contigs in all categories. Download TABLE S1, PDF file, 0.1 MB. Copyright © 2017 Luo et al. 2017 Luo et al. This content is distributed under the terms of the Creative Commons Attribution 4.0 International license.

10.1128/mBio.01903-17.7 TABLE S2  List of 10 out of 129 ALOHA viral contigs with hits to known phages in RefSeq75 database with more than half of its genes hitting an average amino acid identity of >60%. Download TABLE S2, PDF file, 0.1 MB. Copyright © 2017 Luo et al. 2017 Luo et al. This content is distributed under the terms of the Creative Commons Attribution 4.0 International license.

Mesopelagic phages appeared to be undersampled in current databases and were distinct from surface water phages. None of the ALOHA viral contigs from 770 m or deeper were similar to any previously reported phages in existing databases. Gene mapping revealed that some ALOHA viral contigs shared conserved genomic structure with known phages in reference databases, despite low amino acid similarity (Fig. S3a to e), supporting the existence of evolutionary constraints on gene order even among distantly related phages.

10.1128/mBio.01903-17.3 FIG S3  Genome maps of abundant phages found at different depths in the water column at Station ALOHA. Download FIG S3, PDF file, 0.3 MB. Copyright © 2017 Luo et al. 2017 Luo et al. This content is distributed under the terms of the Creative Commons Attribution 4.0 International license.

Among viral contigs of all lengths, we found 37 genes identified from 625 contigs colocated with phage structural genes that have functions not previously identified in marine phages (Table S3). These included 19 putative AMGs that provide insight into how phages can manipulate host metabolism. Some of these genes have putative functions in antibiotic synthesis (carbamoyltransferase C terminus, myoinositol-1-phosphate synthase), antimicrobial resistance (dolichyl-phosphate-mannose protein mannosyltransferase), antitoxin synthesis (antitoxin of toxin-antitoxin system), antigen synthesis (P83/100), transporters (sodium bile acid symporter), and superinfection immunity. These new phage-associated genes further expand our current knowledge of gene content in naturally occurring phages. Moreover, certain AMGs were found only in ALOHA viral contigs dominant at specific depths, such as myoinositol-1-phosphate synthase (25 and 75 m), dolichyl-phosphate-mannose protein mannosyltransferase (200 m), sodium bile acid symporter (200 m), P83/100 antigen proteins (200 m), and superinfection immunity protein (sporadic, 770 and 1,000 m). The functional roles of these depth-specific AMGs provide new insight into phage-host interactions in the open-ocean water column.

10.1128/mBio.01903-17.8 TABLE S3  List of 37 novel genes in marine phages identified from 625 curated contigs colocated with viral structural genes (PFAM bit score of >10) that have not been reported in other previously sequenced viromes (Brum et al., 2015; Roux et al., 2016). Download TABLE S3, PDF file, 0.1 MB. Copyright © 2017 Luo et al. 2017 Luo et al. This content is distributed under the terms of the Creative Commons Attribution 4.0 International license.

### Phage genotype distributions in the Station ALOHA time-series depth profile.

**FIG 2:**
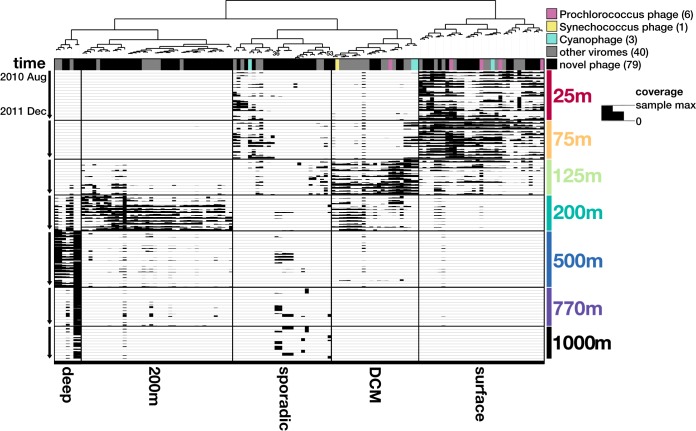
Coverage profiles of 129 ALOHA viral contigs through depth and time. Each node on the top dendrogram and its associated column represent one contig, while each row represents one of 83 total samples. The height of the black column within a cell represents the mean coverage (second and third quartiles) of a contig in a given sample. The full bar height of each sample layer is log scaled to the maximum coverage in a given sample. Sample layers are ordered by depth from 25 to 1,000 m, shown on the right. Within each depth bin, samples are ordered by time from August 2010 to December 2011, shown on the left. Contigs are clustered by differential coverage, with manual binning into five groups on the bottom. Across the top, homology to known phages in the RefSeq75 protein database and other viral metagenomes was assigned based on >50% genes hitting at >60% average amino acid identity.

Most ALOHA viral contigs reached peak abundances at a single depth and could be broadly categorized into one of five groups based on abundance profiles: a surface group dominating 25 to 75 m, a deep-chlorophyll-maximum (DCM) group at 125 m, a 200-m group, a deeper mesopelagic group from 500 to 1,000 m, and a sporadic group of more temporally variable phages (Fig. 2). No evidence for eurybathic phages was evident in our data. Instead, the vertical distribution of phage contigs appeared to reflect the depth-stratified distributions of their potential bacterial and archaeal hosts (26). Overall, these results suggest that many dominant phage groups at Station ALOHA may have relatively narrow host ranges.

**FIG 3:**
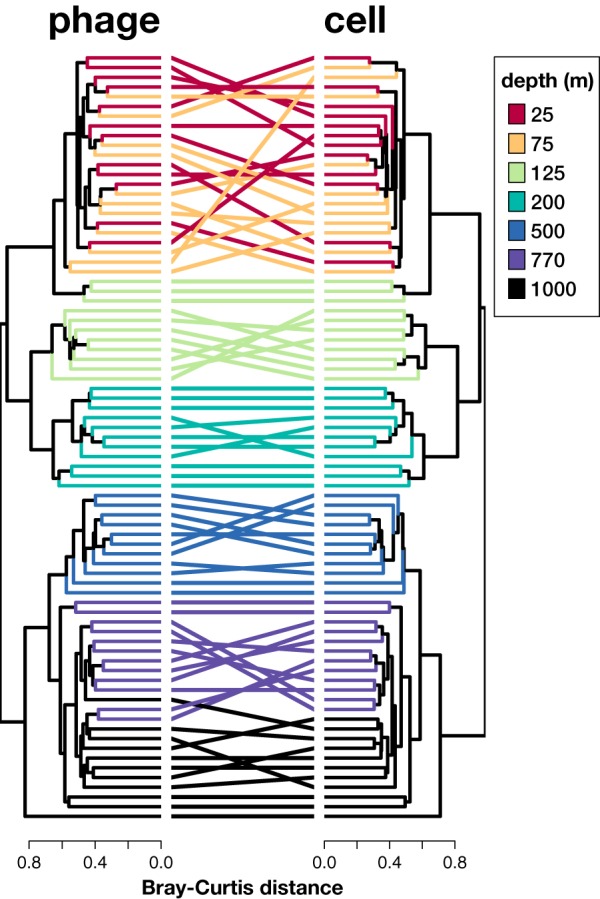
Cluster analysis of phage-specific versus cellular genes identified in the Station ALOHA time-series nonredundant gene catalog. Each edge on the trees represents one sample, and corresponding samples are shown with connecting lines between the two dendrograms.

This depth structure in ALOHA viral contig distributions was also reflected in our gene-centric approach using the ALOHA gene catalog (26). Comparison of sample clustering patterns based on phage- versus bacterioplankton-specific genes indicated that viral gene distributions mirrored community-wide trends of their potential bacterioplankton hosts. Overall patterns of depth-specific gene content were highly similar between phage-specific and cellular genes (Fig. 3), reflecting depth clustering patterns in the genome-centric approach. Phage- and cell-associated gene distribution dendrograms were conserved across these depth clusters over time. This suggested that the overall pattern of gene distributions was conserved between phages and their cellular host community.

**FIG 4:**
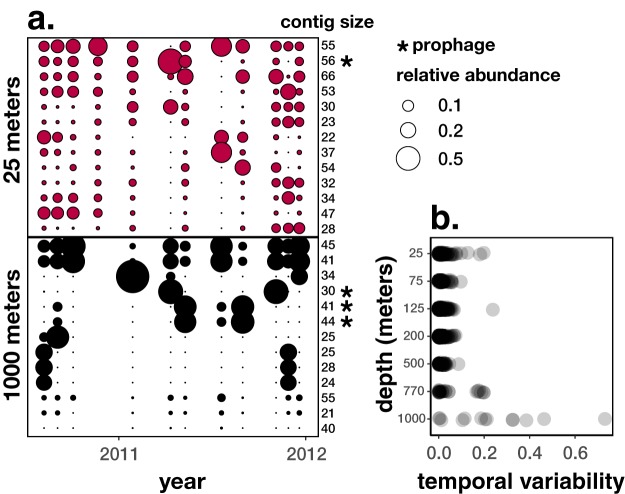
Temporal variability of assembled viral contigs increased with depth. (a) Relative abundance of the most abundant ALOHA viral contigs at 25 and 1,000 m. Each row represents one of the 13 most abundant ALOHA viral contigs for each depth bin, with size indicated in kilobase pairs (kbp). Asterisks represent contigs containing one or more prophage markers (PFAM bit score of >30 to integrase, CI repressor, Cro, or excisionase). Relative abundance is scaled by area. (b) Mean-normalized variance of relative abundance through time calculated for each contig.

The pronounced spatial differences along the depth gradient were accompanied by depth-stratified differences in temporal variability of ALOHA viral contigs, with persistent phages dominating surface waters and more episodic phages dominating mesopelagic depths. Most phages displayed no clear trends of seasonality or shuffling of dominant phage groups, with the exception of a small group of phages in the sporadic group (Fig. 2). In the persistent groups (surface, DCM, 200 m, and mesopelagic), phage populations displayed remarkable temporal stability throughout the 1.5-year sampling period, similar to that previously reported for surface water viruses over shorter daily time scales (24). Additionally, four phages in our persistent group, captured in the 2010–2011 Hawaii Ocean Time-series (HOT) data set, shared high sequence similarity to phages found in a 2015 diel 15-m phage study in the NPSG (24), indicating that these populations were consistently present over multiple years (Table S4). In contrast to the patterns observed in surface waters, phages in the mesopelagic ocean exhibited a more-sporadic occurrence characteristic of boom-and-bust dynamics. Several ALOHA viral contigs were highly abundant at only a few time points and virtually absent at other times, as shown by the relative abundances of phages at 25 and 1,000 m (Fig. 4a). To confirm that the greater temporal variability at 1,000 m is not attributed to the lower number of viral contigs detected, mean-normalized variances show that mesopelagic phages were indeed more temporally variable than surface water phages (Fig. 4b).

10.1128/mBio.01903-17.9 TABLE S4  List of 4 persistent ALOHA viral contigs with hits to surface phages in a 2015 data set near Station ALOHA (Aylward et al., https://doi.org/10.1073/pnas.1714821114) with more than half of its genes hitting an average amino acid identity of >60%. Download TABLE S4, PDF file, 0.1 MB. Copyright © 2017 Luo et al. 2017 Luo et al. This content is distributed under the terms of the Creative Commons Attribution 4.0 International license.

### Genomic trends in lytic versus lysogenic viral life history.

**FIG 5:**
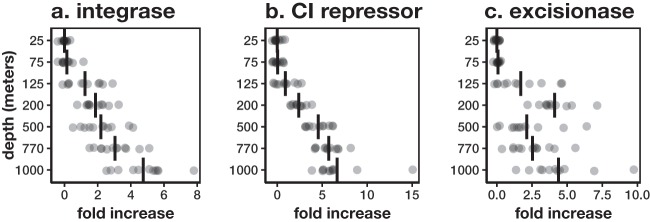
Depth profile of prophage marker proteins identified in the Station ALOHA time-series nonredundant gene catalog (domain bit score of >50) using hidden Markov models generated with manually curated sets of viral marker proteins from NCBI. Each circle represents a sample mean, and each vertical bar represents a depth mean. Depth profile of prophage markers integrase (a), CI repressor (b), and excisionase (c). The fold increase of prophage markers with respect to surface mean is calculated using coverage of marker proteins normalized to coverage of capsid proteins.

The phage depth distributions were also reflected in encoded viral life-history traits. Using prophage marker genes as indicators, we found that the genomic potential for lysogeny increased below the DCM, which hovers around 90 to 130 m throughout the year (25). We focus on three known prophage markers from studies on bacteriophage lambda as proxies for lysogeny, though it is worth noting that this is not the only means of lysogenic conversion. It should be noted that some putative prophage markers also exist in nonphage genetic elements, for example, the integrases associated with integrative conjugative elements. In our analyses, all three prophage markers (integrase, repressor protein cI [CI repressor], and excisionase) showed similar patterns of proportional increase with depth below 125 m (Fig. 5a to c). Collectively, the data suggested that the proportion of phages that are capable of lysogeny was ~5 times higher in the mesopelagic than in the surface ocean. Furthermore, the average number of prophage markers per cellular genome increased below 125 m, suggesting that the average number of integrated prophages per cell also increased with depth (Fig. S4a to c). The copy number of integrases appears to be anomalously high relative to other prophage and phage markers, suggesting that there may be representation from non-phage mobile genomic elements or that these gene families are more highly conserved and therefore more easily detected using homology-based methods. Despite the possibility of false positives in using integrase as a prophage marker, the overall increased abundance of other prophage markers provides evidence for an increased prevalence of lysogeny in the mesopelagic ocean. Lastly, the average copy number of 4 phage markers per cellular genome ranged from 1 to 5 at 125 m while values were 0.45, 0.31, 0.52, and 0.04 at 1,000 m for DNA polymerase, terminase, capsid, and tail, respectively (Fig. S4d to g). The strikingly similar decreases across all four phage markers suggest that the prevalence of actively replicating lytic phages within cells decreased greatly at mesopelagic depths. The potential inclusion of free phages adhered to particles in our samples is unlikely to significantly impact these results due to prefiltration of larger, >1.6-μm particles before bacterioplankton collection. Taken together, these marker gene data suggest a shift of increased lysogeny and overall decreased viral particle abundance per host, from the euphotic to the mesopelagic zone.

10.1128/mBio.01903-17.4 FIG S4  Depth profile of phage marker proteins identified in the Station ALOHA nonredundant gene catalog (domain bit score of >50) using hidden Markov models generated with a manually curated set of proteins from NCBI. Download FIG S4, PDF file, 0.2 MB. Copyright © 2017 Luo et al. 2017 Luo et al. This content is distributed under the terms of the Creative Commons Attribution 4.0 International license.

Viral life-history strategies are important to consider for both phage and host ecology. For the host, a lytic cycle results in cell death and the release of cellular material into the environment, while a lysogenic cycle does not immediately kill the host but incurs a cost of carrying and reproducing foreign genetic material in the host genome. For the phage, a lytic cycle means a rapid increase in short-term fitness when a host is productive enough to support phage production, while lysogeny may represent an opportunity cost in reproduction but increased chances of survival. Characterizing which environments favor a certain viral life-history strategy is important when considering virus-host interactions and their resulting biogeochemical effects along the ocean’s interior.

Different theories exist to explain the ecologic factors that may influence the proportion of lytic versus lysogenic phages. For example, the “piggy-back the winner” hypothesis predicts that lysogeny should be more prevalent at higher host cell densities (28) and has been based on cell- and virus-like particle abundance patterns (9, 28, 29). Our metagenomic data do not directly support these results, since we found evidence for more lysogens in deeper waters that have lower cell densities (cell abundance data available at http://hahana.soest.hawaii.edu/hot/hot-dogs/), consistent with earlier results from mitomycin C phage induction studies (30).

An alternative hypothesis is that lysogeny will be favored in environments where there are fewer constraints on cellular genome size (31, 32). In surface oligotrophic oceans, bacteria tend to have smaller genomes, which has been hypothesized to be a consequence of low-nutrient conditions leading to metabolic constraints that favor smaller genomes, lower G+C contents, and decreased nitrogen content in peptides (25, 33). At Station ALOHA, bacterial genome size nearly doubles just below the DCM in the “genomic transition zone” (26), where we also observed a marked increase in prophage marker genes. This increase in genome size below the DCM is likely more permissive to foreign DNA accumulation, thereby enabling replicative elements such as prophages to persist in the more capacious bacterial genomes that comprise deeper-water microbial communities.

Lysogeny might also be advantageous in the deep ocean where low host productivity cannot support rapid phage production (34–36). The results reported here are consistent with a prior prophage induction experiment that showed greater potential for lysogeny in deep than in surface waters in the Mediterranean and Baltic Seas (30). Other field observations have found increased lysogeny in low-productivity environments such as oligotrophic rather than coastal oceans (37, 38) or in winter rather than spring (23, 39). Although this trend is not consistent across all studies (e.g., references 40 and 41), productivity appears to be a major correlate of phage-host interactions in some environments. At Station ALOHA, productivity declines sharply past 125 m near the DCM (25), coinciding with the sharp transition from lytic to lysogenic phage-host interaction in our marker gene profiles. Our study provides a genomic perspective and validation for induction experiments, as well as molecular evidence for a higher proportion of lysogens and decreased viral particles per host in the deep ocean. Our results also suggest that phage-induced mortality may be higher in more productive surface waters, shifting to more temperate phage-host interactions in the mesopelagic open ocean.

### Ecology of surface and mesopelagic phages.

Overall, the most abundant surface phage populations were well represented in the 1.6- to 0.2-μm size fraction throughout our sampling period (Fig. 2 and 4). This temporal persistence is intriguing, since dominant phages are expected to follow boom-and-bust dynamics in a negative density-dependent manner, according to some ecological models such as kill-the-winner or fluctuating selection dynamics model (reviewed in reference 42). On the other hand, phage resistance mechanisms allow coastal marine *Synechococcus* populations to coexist with abundant cyanophages over seasonal cycles (18), and persistent viral types have been observed in other viral time-series analyses (19–22). Moreover, the stability of phages in surface waters may reflect the overall stability of prokaryotic assemblages in the NSPG (43). Alternatively, phages could display increased temporal persistence due to a broader host range or longer viral residency time. A combination of these factors might contribute to the temporal stability of many different host-phage pairs, particularly those persistent in ocean surface waters.

The most temporally variable phage groups in our analyses occurred in the mesopelagic ocean, where specific phages dominate at certain time points only to disappear the next month (Fig. 2 and 4). This sporadic nature corresponds to high temporal variability in known phage and bacterial genes observed in the mesopelagic ocean, particularly at 1,000 m (Fig. S5a and b). Compared to the surface ocean, where sunlight drives consistent high productivity, productivity in the deep ocean is limited by sporadic rain of organic material exported from the surface (25). Temporally variable resources might select for copiotrophic or particle-attached bacteria (44) that can grow quickly when resources become available. These bursts of growth can lead to temporally sporadic phage distributions from prophage propagation with fast-growing hosts, prophage induction, or particularly successful lytic infections in terminating host blooms.

10.1128/mBio.01903-17.5 FIG S5  Bubble plot of proportion of ALOHA gene catalog genes hitting four groups of known phages (a) and associated bacteria (b). Download FIG S5, PDF file, 0.3 MB. Copyright © 2017 Luo et al. 2017 Luo et al. This content is distributed under the terms of the Creative Commons Attribution 4.0 International license.

In this environment of temporally variable resources, we found one novel gene of interest that is specific to a mesopelagic sporadic phage: a superinfection immunity gene encoding a membrane-attached protein that confers host immunity to other phages (45) that has not been previously observed in marine phages. The associated contig (Fig. S3e) in our sampling period followed the distribution of *Vibrio* (Fig. S5b), copiotrophic bacteria that have been found on sinking particles and in deep samples at Station ALOHA (46). This putative lytic vibriophage is remarkably abundant in an environment where lysogeny is favored. By conferring host immunity to other competitor phages, this gene could contribute to the success of this abundant putative lytic phage and result in subsequent boom-and-bust dynamics of this phage and its particle-attached host in the mesopelagic ocean. It is worth noting here that such genomic characterization of phage populations *in situ* may be prone to generating false positives in functional capacity (47). Experimental verification of the function of carried phage genes will more reliably elucidate the genomic capacity of previously uncharacterized mesopelagic phages and the resulting phage-host interaction.

### Conclusions.

In summary, our time-series study in the NPSG leveraged both gene- and genome-centric approaches to provide insight into phage diversity, structure, and function across depth and time. We found that mesopelagic phages were distinct from surface phages and were largely novel and underrepresented in current viral reference and metagenomic databases. With respect to depth variability, discrete phage populations displayed strong depth structure, similar to that of putative bacterial hosts. There were virtually no eurybathic phages, suggesting that most dominant phage groups were adapted to relatively narrow host ranges. We also found unique AMGs in a mesopelagic phage suggestive of depth-specific adaptations to a more variable landscape of phage-host interactions in the aphotic zone. With respect to temporal variability, the most abundant phage groups were remarkably persistent, displaying little to no seasonality nor observable shuffling of dominant groups. With respect to coupled variability through space and time, mesopelagic phages were more sporadic in their distribution. Considering viral life history, we found five times more genes associated with lysogeny at greater depths and a sharp increase in lysogeny at and below the DCM, corresponding to the cell-associated genomic transition zone (26). Our observations, in addition to other recent studies (13–17), expand the realm of characterized phages in the surface to the mesopelagic ocean.

## MATERIALS AND METHODS

### Study site and sample collection.

Bacterioplankton samples in the 0.2- to 1.6-µm size fraction were collected from 7 depths (25, 75, 125, 200, 500, 770, and 1,000 m) on 12 occasions in 2010–2011 at Station ALOHA (22°45′N, 158°W) in the North Pacific Subtropical Gyre (NPSG). Detailed sample collection has been previously described (26). As the study site of the Hawaii Ocean Time-series (HOT) program, Station ALOHA is one of the most sampled open-ocean systems in the world, with well-characterized biogeochemical gradients that provide context to our work (25) (examples in Fig. S1 in the supplemental material).

### Station ALOHA metagenomic assembly and gene catalog.

The methods for DNA extraction, library construction, and sequencing with the Illumina NextSeq and MiSeq platforms of these bacterioplankton samples have been previously described in detail (26). Briefly, metagenomic reads from each sample were assembled using MIRA v4.9.5_2, providing the basis for our genome-centric analyses. Forty million genes were predicted from all assemblies using Prodigal v2.6.0 (48) and clustered using CD-HIT v2.6 (49) to generate 8,966,703 nonredundant gene clusters. These nonredundant genes are here referred to as the ALOHA gene catalog, providing the basis for our gene-centric analyses. The relative abundance of each nonredundant gene in each sample was calculated by mapping reads using the BWA-MEM algorithm v0.7.15 (50) with default parameters and then dividing the resulting coverage by the total coverage of all genes (26).

### Genome-centric analyses. (i) Assembling ALOHA viral contigs.

Station ALOHA metagenomic assemblies were used to identify viral contigs (Fig. S2) using VirSorter v1.0.3 with a 3-kbp cutoff for improved recall (51). Sequence reads that were used to assemble these contigs were pooled across all samples and reassembled using default parameters in metaSPAdes (52). The 104,732 contigs were validated through a second VirSorter screen, in which 917 viral contigs 1.7 to 108 kbp in length from all VirSorter categories were retained. Most cellular contamination was removed at this stage, as the estimated cellular genomic completeness was only 1.2%, with only 35 out of 16,296 genes mapping to single-copy prokaryotic marker genes using the standard Anvi’o v.2.1.1 workflow (53–55). Of these 35 hits, 30 were restricted to *recA* and DNA helicase, which could be recombination and replication homologous phage genes. One contig found to contain a ribosomal bacterial marker gene was removed from our analyses.

Given that 20 kbp represents the lower end of genome size in DNA phages from marine systems (56), we retained 142 contigs of ≥20 kbp in size to focus on near-complete genomes or large genomic fragments. As a final quality control step, we removed contigs that did not contain any genes with distant homology to known phage structural proteins (PFAM bit score of >10 to terminase, portal, capsid, tail, base plate, spike, neck, and head genes). This step was conservative and eliminated 13 putative phage genomes from our analyses, 5 of which displayed significant homology to previously sequenced phages (identification described below). The resulting contigs represent a high-confidence subset of total phage diversity. We checked again for cellular contamination using cell genomic completion, which is now reduced to 0.6%. Only 12 out of a total of 5,877 putative phage genes mapped to bacterial or archaeal marker genes. Eleven of these hits are restricted to *recA* and DNA helicase, while none were ribosomal cell marker genes. Overall, these quality control steps successfully removed any detectable cellular genome contaminants, generating 129 ALOHA high-confidence viral contigs ranging between 20 and 108 kbp in length. Assembly and reassembly statistics are shown in Table S1.

### (ii) Annotating ALOHA viral contigs.

Contigs were annotated with a combined viral database from predicted proteins in assembled sequences from phages in RefSeq75 (57) and four other viral metagenomes: uvMED (15), uvDeep (17), GOV (14), and EV (27). Genes were predicted using Prodigal v2.6.3 (48). Contigs were identified using LAST (58) to this combined viral database with cutoffs of >50% of genes hit to a reference with >60% average amino acid identity. This relaxed cutoff is chosen to identify distant homology to limited reference phages based on a majority of genes on a given contig. To calculate the proportion of genomes assigned to each category in a given sample, contigs were normalized by their relative abundance of nucleotides mapped.

### (iii) Depth and temporal distributions of ALOHA viral contigs.

For reference-independent visualization of spatiotemporal distributions, we used BWA-MEM v0.7.15 (50) to map all reads from each sample to ALOHA viral contigs and generated coverage profiles using Anvi’o v.2.1.1 (55). To prevent the possibility of conserved phage genes inflating coverage, only the second and third coverage value quartiles across each contig were used to generate mean coverage profiles. ALOHA viral contigs were binned by differential coverage manually into five distribution groups. To examine temporal variability within each depth bin, we used the proportion of nucleotides mapped as a metric for relative abundance and plotted the 13 most abundant phages through time within each depth bin. To examine whether temporal variability in phage assemblages changes with depth, we calculated the variance of the relative abundance for each ALOHA viral contig within all depth bins through time. Given that more abundant contigs tend to have larger variances, we normalized the temporal variance of each contig by dividing by its mean abundance through time. This mean-normalized variance alleviates preferential weighting to high-abundance contigs and prevents changes in number of contigs among depths from influencing the variance metric.

### (iv) Mapping to reference genomes.

To examine the genomic structure of our ALOHA viral contigs, we generated genome maps of some of the most abundant or complete ALOHA viral contigs in each of five depth distribution groups. Phage genomes in the RefSeq75 database that were most similar to each ALOHA viral contig were included to visualize structural similarities using the GenomeDiagram module v0.2 in Python (59).

### (v) Detection of novel phage genes and AMGs.

To detect AMGs, we predicted and annotated 16,286 genes from 917 reassembled viral contigs (all lengths) and compared these genes to existing databases of marine phage genes including AMGs (13, 14). Genes were considered novel AMGs if they were both annotated with a PFAM bit score of >30 to a novel function not in these databases and colocated on the subset of 625 contigs with viral structural genes (PFAM bit score of >10 to terminase, portal, capsid, tail, base plate, spike, neck, and head).

### Gene-centric analyses. (i) Phage- and cell-associated gene catalog assemblages.

We clustered samples using the ALOHA gene catalog to examine how closely patterns of spatiotemporal diversity of phage assemblages mirrored those of broader bacterioplankton communities. We separated genes into two groups of interest: a phage group of 177,713 viral genes and a cell-associated group encompassing all remaining genes in the ALOHA gene catalog (8,788,990 in total). Viral genes were identified using the combined viral database described above with LAST at >90% amino acid identity. A total of 5,328 photosystem genes (PFAM bit score of >30) were removed from the phage group on the grounds that phage copies of this AMG (reviewed in reference 60) could not be accurately distinguished from bacterium-encoded copies (3). This approach identified 172,385 phage genes used in subsequent analyses. Independently for phage- and cell-associated groups, we clustered samples using gene coverages to generate Bray-Curtis distance matrices and subsequently average linkage hierarchical agglomerative clustering on the R vegan package (61). The resulting dendrograms were visualized using the R dendextend package (62).

To visualize the depth structure and distribution of known phages and bacteria, we identified 180,055 genes from the ALOHA gene catalog with >60% amino acid identity to known phages and 3,295,154 bacterial genes with >60% amino acid identity to known bacteria in RefSeq75. Using these gene coverages, we calculated relative abundances of three groups of well-characterized phages and their associated bacterial hosts for each sample.

### (ii) Viral life history.

To examine whether the prevalence of lysogeny changes with depth, we identified prophage markers in the ALOHA gene catalog and used their relative abundance as a proxy for the potential incidence of lysogeny. We also analyzed other sets of well-known phage structural proteins for normalization. We curated sets of annotated prophage and phage markers of interest from NCBI and identified 89 prophage integrases, 116 prophage CI repressors, 357 prophage excisionases, 1,780 phage DNA polymerases, 139 phage terminases, 971 phage capsids, and 104 phage tail fiber proteins. Curated proteins were selected from either full phage genomes or uncultured marine phages from previously sequenced viral metagenomes. We used these protein sets to generate hidden Markov models for each marker using MUSCLE v3.8.31 alignment and HMMER v3.1 (63, 64). We then used these models to identify marker proteins in the translated ALOHA gene catalog with a domain bit score of >50 across the whole sequence. The summed coverages of prophage markers (integrases, CI repressors, and excisionases) were normalized to the summed coverage of capsids as a proxy for the proportion of phages that had the potential to reproduce through lysogeny based on the presence of prophage marker genes. We further normalized this proportion by dividing by the surface ocean mean value to identify relative changes in lysogeny with depth.

To examine depth profiles of the number and nature of active infections, we calculated the average cell genomic copy number of phage markers (DNA polymerases, terminases, capsids, and tail fibers) and prophage markers (integrases, CI repressors, and excisionases). Given that prophages in cellular genomes are predominantly nonreplicative, the copy number of prophage markers was used to estimate the number of prophages per genome. The copy number of phage markers was used to estimate the number of total phages captured inside a bacterium. To generate copy number per cellular genome, coverages of prophage and phage markers were normalized to the average coverage of 10 universal single-copy bacterial marker genes, called mOTU profiling (26, 65).

### Accession number(s).

Sequence data are available at NCBI under BioProject no. PRJNA352737 and at https://www.imicrobe.us/#/projects/263.
