# Supplementary material for: Bacteriophage Distributions and Temporal Variability in the Ocean’s Interior
Source: mBio. 2017 Nov 28;8(6):e01903-17. doi: 10.1128/mBio.01903-17 (PMC5705922; doi:10.1128/mBio.01903-17)

Supplementary Figure 1. Our dataset includes 83 samples from 7 depths and 12 time points from August 2010 to December 2011. This sampling captures in-situ assemblages along sharp biogeochemical gradients such as a) temperature, b) nutrients, and c) chlorophyll fluorescence showing the deep chlorophyll maximum at 90-130m.

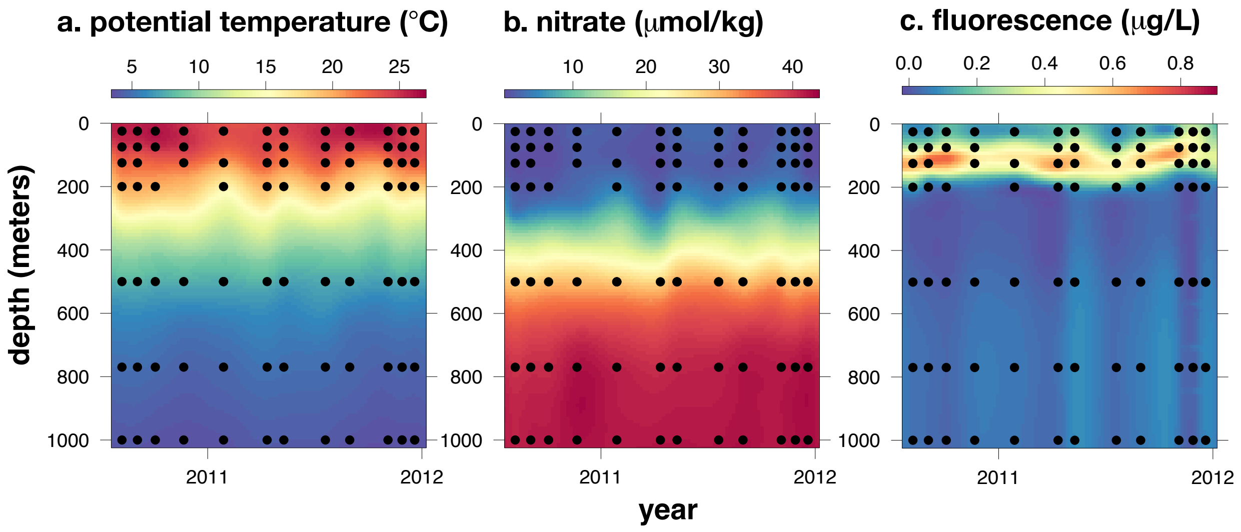

Supplement: FIG S1 [file mbo006173616sf1.pdf]
