# Supplementary material for: Bacteriophage Distributions and Temporal Variability in the Ocean’s Interior
Source: mBio. 2017 Nov 28;8(6):e01903-17. doi: 10.1128/mBio.01903-17 (PMC5705922; doi:10.1128/mBio.01903-17)

Supplementary Figure 2. Bioinformatic workflow from sequencing to gene- and -centric approaches. Analyses presented in main and supplementary figures are highlighted with black backgrounds. The original Station ALOHA catalogue used to form the basis for this work was reported in Mende et al, 2017.

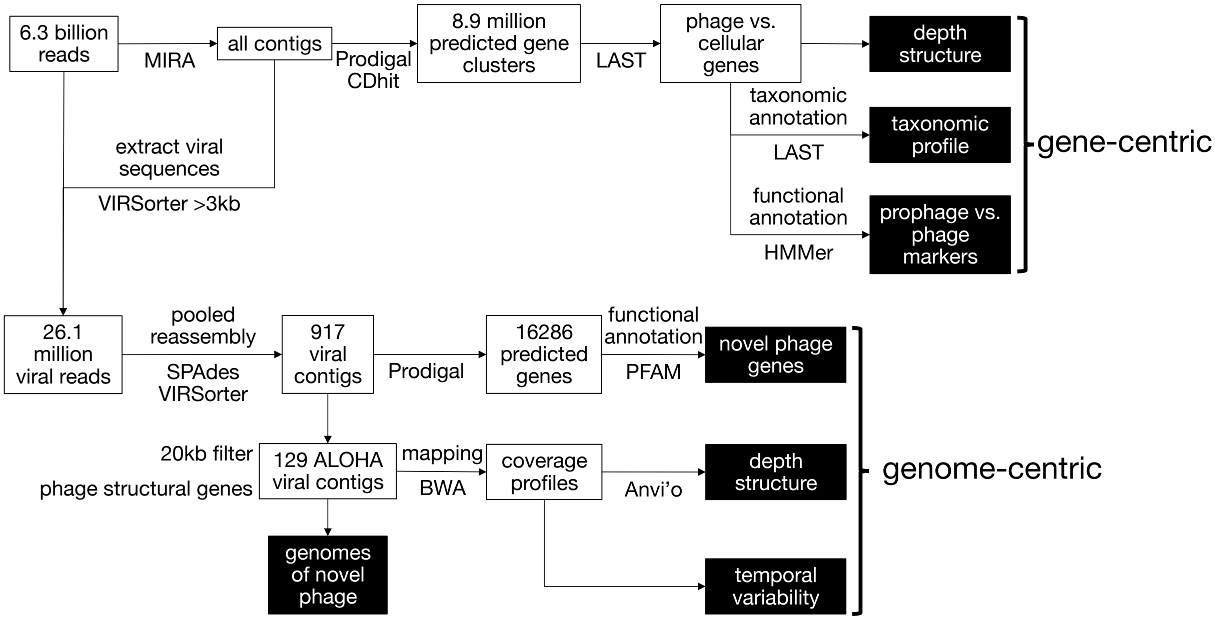

Supplement: FIG S2 [file mbo006173616sf2.pdf]
