# Supplementary material for: Bacteriophage Distributions and Temporal Variability in the Ocean’s Interior
Source: mBio. 2017 Nov 28;8(6):e01903-17. doi: 10.1128/mBio.01903-17 (PMC5705922; doi:10.1128/mBio.01903-17)
Supplement: TABLE S1 [file mbo006173616st1.pdf]

Supplementary Table 1. Assembly statistics for the initial individual assemblies for each sample, number of contigs >3kb in length used for VIRSorter runs, and number of VIRSorter identified contigs in all categories. Reads from these VIRSorter contigs were then pooled to generate the viral reassembly, which passed through additional quality-control steps to generate final ALOHA viral contigs.

**initial individual assemblies**								**pooled viral reassembly**			
**cruise**	**seq. run**	**depth**	**all reads**	**all contigs**	**>3kb contigs**	**VIRSorter contigs**	**VIRSorter reads**	**all contigs**	**VIRSorter contigs**	**>20kb filter**	**ALOHA viral contigs**
HOT224	1	25	68795546	780250	8676	998	542961	104732	917	142	129
HOT224	1	45	38277419	436530	8648	330	141926				
HOT224	1	75	62434378	675786	7264	943	484334				
HOT224	1	125	33557304	327780	8778	404	246891				
HOT224	1	200	27557654	164297	1222	19	21808				
HOT224	1	500	39143776	329910	7135	67	41532				
HOT224	1	770	39044958	387925	10488	45	17836				
HOT224	1	1000	50259388	496232	13460	135	115413				
HOT225	1	25	35576272	434504	9755	513	182372				
HOT225	1	45	58874846	720091	7768	333	114849				
HOT225	1	75	50099672	579167	9528	1076	657787				
HOT225	1	125	37740015	269950	2871	160	84479				
HOT225	1	200	32974908	262614	2533	46	37615				
HOT225	1	500	61257440	592008	15445	236	167380				
HOT225	1	770	37462326	379836	10670	53	22013				
HOT225	1	1000	37018306	348456	8147	58	25284				
HOT226	1	25	38545192	246479	3661	138	68042				
HOT226	1	45	38860404	418488	5588	265	135878				
HOT226	1	75	37924780	242771	7469	192	69443				
HOT226	1	200	29131958	151367	452	9	2514				
HOT226	1	500	27763940	198519	1447	6	2712				
HOT226	1	770	23025498	179291	1523	2	859				
HOT226	1	1000	34099068	285546	2835	3	963				
HOT227	1	25	31015782	160109	8453	67	27017				
HOT227	1	45	54588576	333911	2452	113	27689				
HOT227	1	75	20863908	104941	4773	41	14749				
HOT227	1	125	58673416	563141	12251	566	561542				
HOT227	1	770	29684950	248958	1637	9	3600				
HOT229	1	25	15866300	151335	14446	1247	148135				
HOT229	1	125	68900144	536390	4754	181	144104				
HOT229	1	200	219892566	2598365	62364	534	1205969				
HOT229	1	500	94020722	525644	8406	62	85366				
HOT229	1	1000	81638699	743487	10987	33	275403				
HOT229	2	25	46264924	448514	6346	445	280803				
HOT229	2	500	94395654	907741	24310	304	162775				
HOT229	2	770	110472810	1149978	34053	173	67922				
HOT229	3	500	47246262	656552	85902	1535	174339				
HOT231	1	25	2758328	21169	4442	31	1556				
HOT231	1	75	65988470	617360	4499	443	218226				
HOT231	1	125	1954188	5041	54	2	111				
HOT231	1	200	53963094	420162	4373	123	145983				
HOT231	1	500	63035510	490556	7473	249	216338				
HOT231	1	770	62847914	545445	10316	54	20754				
HOT231	1	1000	75332383	660830	9055	125	63468				
HOT231	2	25	64708052	651261	5701	325	102905				
HOT231	2	125	62858612	496745	6291	930	634978				
HOT232	1	25	101135393	1045538	8273	443	308793				
HOT232	1	75	66624258	677497	8651	792	286337				
HOT232	1	125	79244750	741083	9391	306	375192				
HOT232	1	200	179804296	2044277	40804	1075	1010794				

HOT232	1	500	78403226	380410	3664	42	36786
HOT232	1	770	95739381	937124	27556	361	112580
HOT232	1	1000	71620066	645586	8183	39	47363
HOT232	2	500	91876072	843945	18793	175	137943
HOT232	3	500	43586734	581707	79231	857	123812
HOT233	1	25	1932956	16739	2971	23	1031
HOT233	1	75	60599405	488917	6189	616	116916
HOT233	1	1000	75101586	725780	14948	114	52105
HOT233	1c	125	54516104	505293	9647	0	0
HOT233	1c	200	53184084	410387	4045	236	229104
HOT233	1c	770	60375417	539000	12133	87	34297
HOT233	2	25	68079640	766887	10882	488	216266
HOT233	2c	500	62438198	575439	17005	72	47527
HOT234	1	75	60360120	631574	11038	1065	1515852
HOT234	1	200	71289593	532716	7729	578	867680
HOT234	1	500	66557784	570637	12360	148	95943
HOT234	1	770	59799400	497390	8776	187	81637
HOT234	1	1000	42757246	385592	3853	19	15480
HOT234	2	25	5813396	63467	13061	400	46490
HOT234	2	75	6142242	68922	12892	732	147041
HOT234	2	125	68391236	459730	5263	849	2578816
HOT234	2	200	7099726	51322	2508	79	31798
HOT234	3	25	69587030	732288	13601	973	872133
HOT236	1	25	54113650	477890	6200	483	103286
HOT236	1	75	112435135	1320009	8069	105	173282
HOT236	1	125	85336087	738209	5257	168	115681
HOT236	1	200	117076925	1025243	17511	327	285229
HOT236	1	500	154947358	751276	8176	208	785016
HOT236	1	1000	64887254	472905	6114	132	601922
HOT236	2	500	140048230	1171819	25399	1173	1567817
HOT236	2	770	128566144	1236465	32220	458	373421
HOT237	1	25	7543668	61651	8704	85	3896
HOT237	1	75	6314039	67564	11563	129	7214
HOT237	1	200	78611890	664892	5875	129	111294
HOT237	1	500	43359446	614893	97266	452	54946
HOT237	1	770	78187908	805648	22673	62	26423
HOT237	1	1000	129459766	1122956	25169	89	44163
HOT237	2	75	74007793	783100	9990	284	129515
HOT237	2	500	43186099	610238	95821	464	56750
HOT237	2	770	15275186	216918	26698	57	4197
HOT237	3	25	81138274	877747	11541	160	62565
HOT237	3	125	63309805	441548	5095	121	78069
HOT237	3	500	53091295	447697	8176	10	3692
HOT238	1	25	58927359	489829	3209	394	257492
HOT238	1	75	49096904	450292	6696	540	307569
HOT238	1c	125	96914858	815738	16870	1565	2188893
HOT238	1c	200	65735346	601297	7582	264	268898
HOT238	1	500	109166692	552739	5746	111	277537
HOT238	1	1000	102565462	362416	4177	22	35941
HOT238	2	500	101038902	935636	19761	689	473122
HOT238	2	770	126791415	1169739	27823	1672	516841
total			6309588541	57151033	1391529	34732	26073010
