# Supplementary material for: Bacteriophage Distributions and Temporal Variability in the Ocean’s Interior
Source: mBio. 2017 Nov 28;8(6):e01903-17. doi: 10.1128/mBio.01903-17 (PMC5705922; doi:10.1128/mBio.01903-17)
Supplement: TABLE S2 [file mbo006173616st2.pdf]

Supplementary Table 2. List of 10 out of 129 ALOHA viral contigs with hits to known phage in RefSeq75 database with more than half of its genes hitting an average amino acid identity of >60%.

contig	RefSeq75 reference genome	proportion gene hits	AAI %
AVC002	Prochlorococcus_phage_P-SSM2	0.519480519	71.77225
AVC004	Prochlorococcus_phage_MED4-213	0.947368421	79.60166667
AVC031	Synechococcus_phage_ACG-2014j	0.526315789	61.353
AVC038	Cyanophage_P-TIM40	0.666666667	62.78642857
AVC045	Prochlorococcus_phage_P-SSM7	0.518518519	67.07642857
AVC047	Prochlorococcus_phage_P-SSP7	0.638297872	69.564
AVC059	Prochlorococcus_phage_P-SSP7	0.538461538	73.25047619
AVC086	Cyanophage_P-TIM40	0.885714286	81.10741935
AVC119	Prochlorococcus_phage_P-GSP1	0.619047619	69.28615385
AVC140	Cyanophage_P-RSM6	0.949494949	88.15542553
