# Supplementary material for: Bacteriophage Distributions and Temporal Variability in the Ocean’s Interior
Source: mBio. 2017 Nov 28;8(6):e01903-17. doi: 10.1128/mBio.01903-17 (PMC5705922; doi:10.1128/mBio.01903-17)
Supplement: FIG S3 [file mbo006173616sf3.pdf]

Supplementary Figure 3. Genome maps of abundant phages from found at different depths in the water column at Station ALOHA: a) surface, b) DCM, c) 200m, d) mesopelagic, and e) sporadic group shown in Fig. 2. ALOHA viral contigs are displayed on top, while reference genomes of the most closely related phage (most common hit at any amino acid identity) are displayed below. Arrows represents predicted genes, which are color-coded by function. Novel AMG’s are annotated in red text. Blue shading between ALOHA viral contig and reference genome genes represent animo acid similarities of LAST hits. The start and end of genomes are displayed in number of base pairs. Some longer reference genomes have been truncated for clarity.

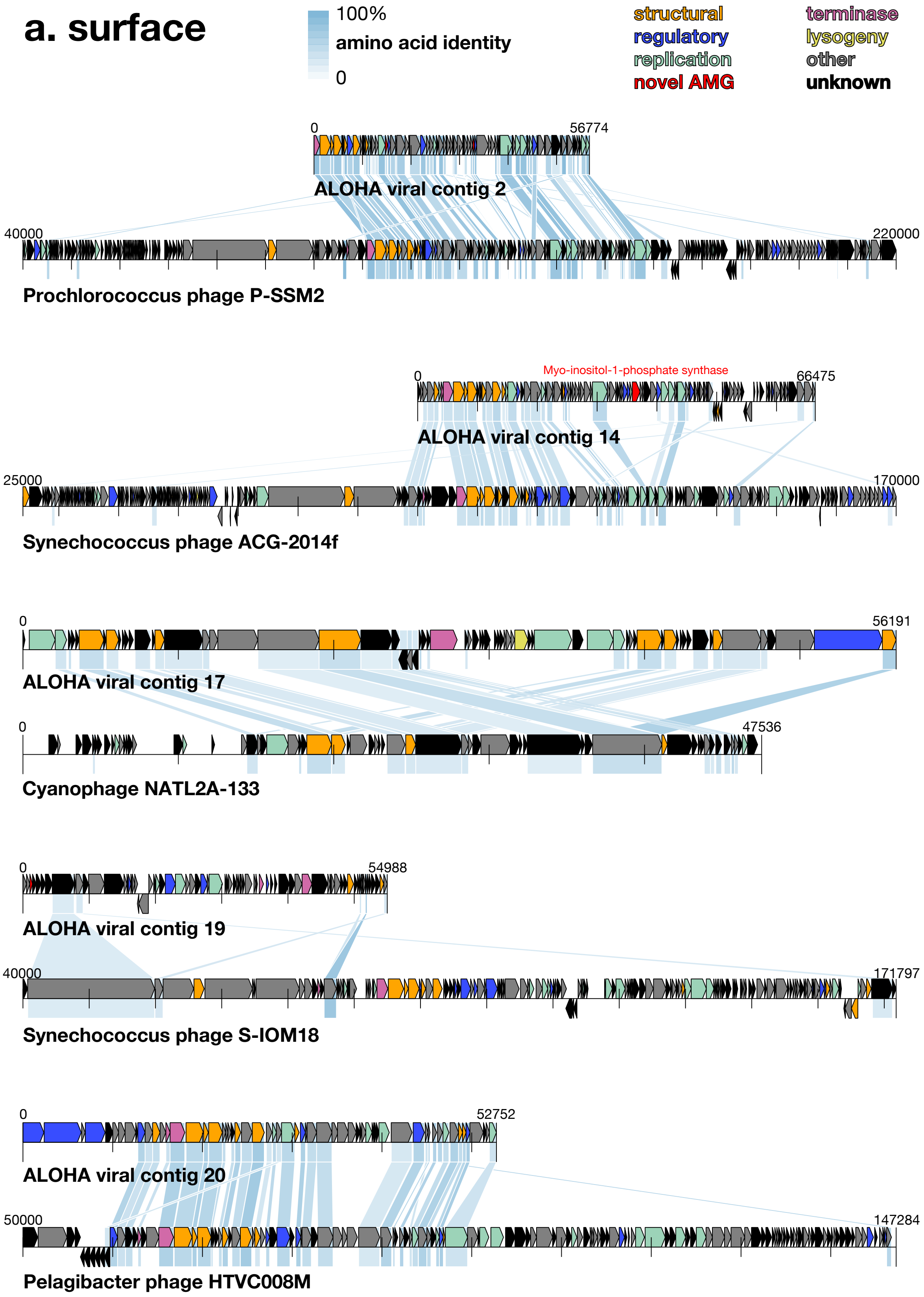
a. surface
100%
amino acid identity
0
structural
regulatory
replication
novel AMG
terminase
lysogeny
other
unknown
0
56774
ALOHA viral contig 2
40000
220000
Prochlorococcus phage P-SSM2
Myo-inositol-1-phosphate synthase
0
66475
ALOHA viral contig 14
25000
170000
Synechococcus phage ACG-2014f
0
56191
ALOHA viral contig 17
0
47536
Cyanophage NATL2A-133
0
54988
ALOHA viral contig 19
40000
171797
Synechococcus phage S-IOM18
0
52752
ALOHA viral contig 20
50000
147284
Pelagibacter phage HTVC008M

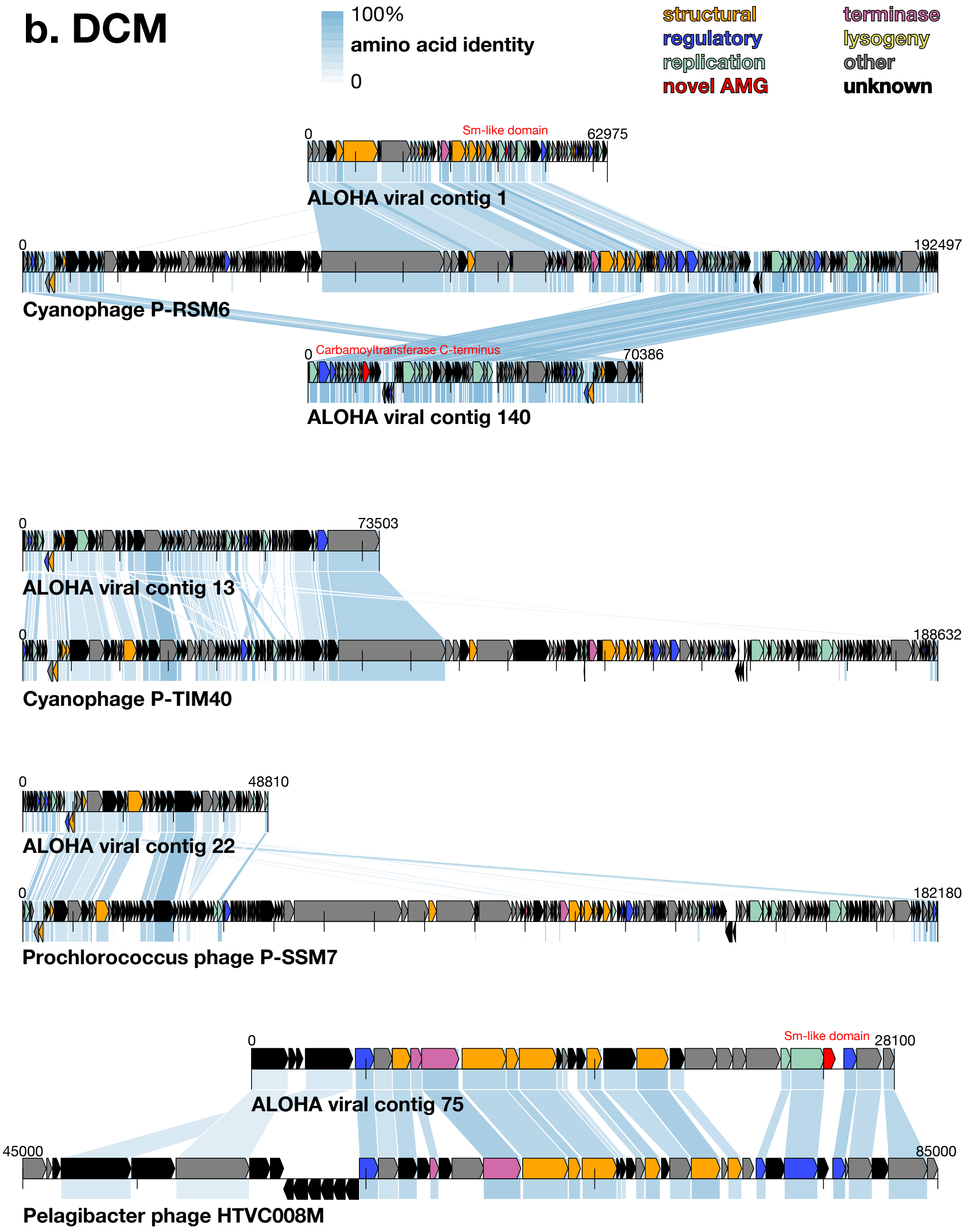
b. DCM
100%
amino acid identity
0
structural
regulatory
replication
novel AMG
terminase
lysogeny
other
unknown
0
Sm-like domain
62975
ALOHA viral contig 1
0
192497
Cyanophage P-RSM6
0
Carbamoyltransferase C-terminus
70386
ALOHA viral contig 140
0
73503
ALOHA viral contig 13
0
188632
Cyanophage P-TIM40
0
48810
ALOHA viral contig 22
0
182180
Prochlorococcus phage P-SSM7
0
Sm-like domain
28100
ALOHA viral contig 75
45000
85000
Pelagibacter phage HTVC008M

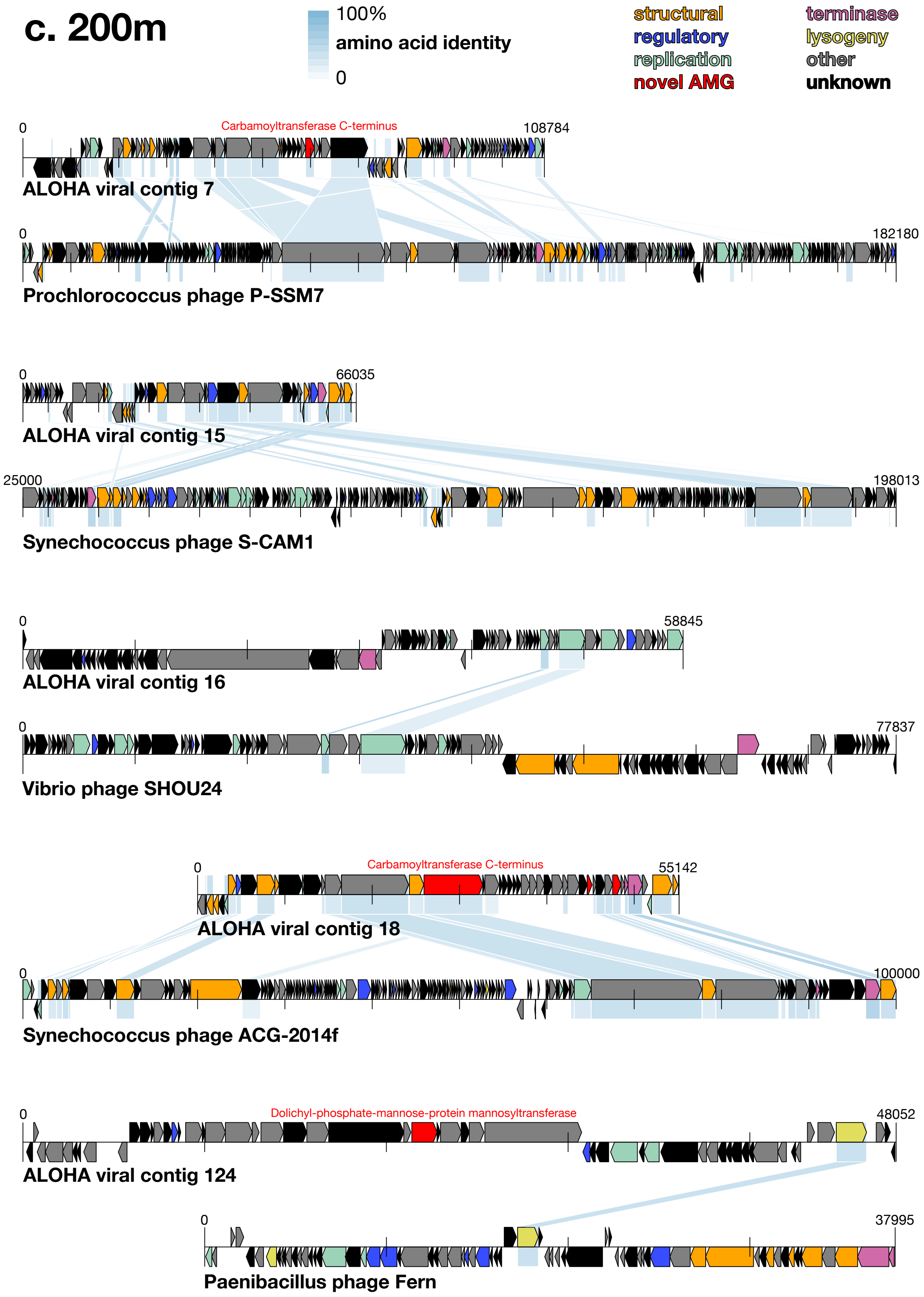
c. 200m
100%
amino acid identity
0
structural
regulatory
replication
novel AMG
terminase
lysogeny
other
unknown
0
Carbamoyltransferase C-terminus
108784
ALOHA viral contig 7
0
182180
Prochlorococcus phage P-SSM7
0
66035
ALOHA viral contig 15
25000
198013
Synechococcus phage S-CAM1
0
58845
ALOHA viral contig 16
0
77837
Vibrio phage SHOU24
0
Carbamoyltransferase C-terminus
55142
ALOHA viral contig 18
0
100000
Synechococcus phage ACG-2014f
0
Dolichyl-phosphate-mannose-protein mannosyltransferase
48052
ALOHA viral contig 124
0
37995
Paenibacillus phage Fern

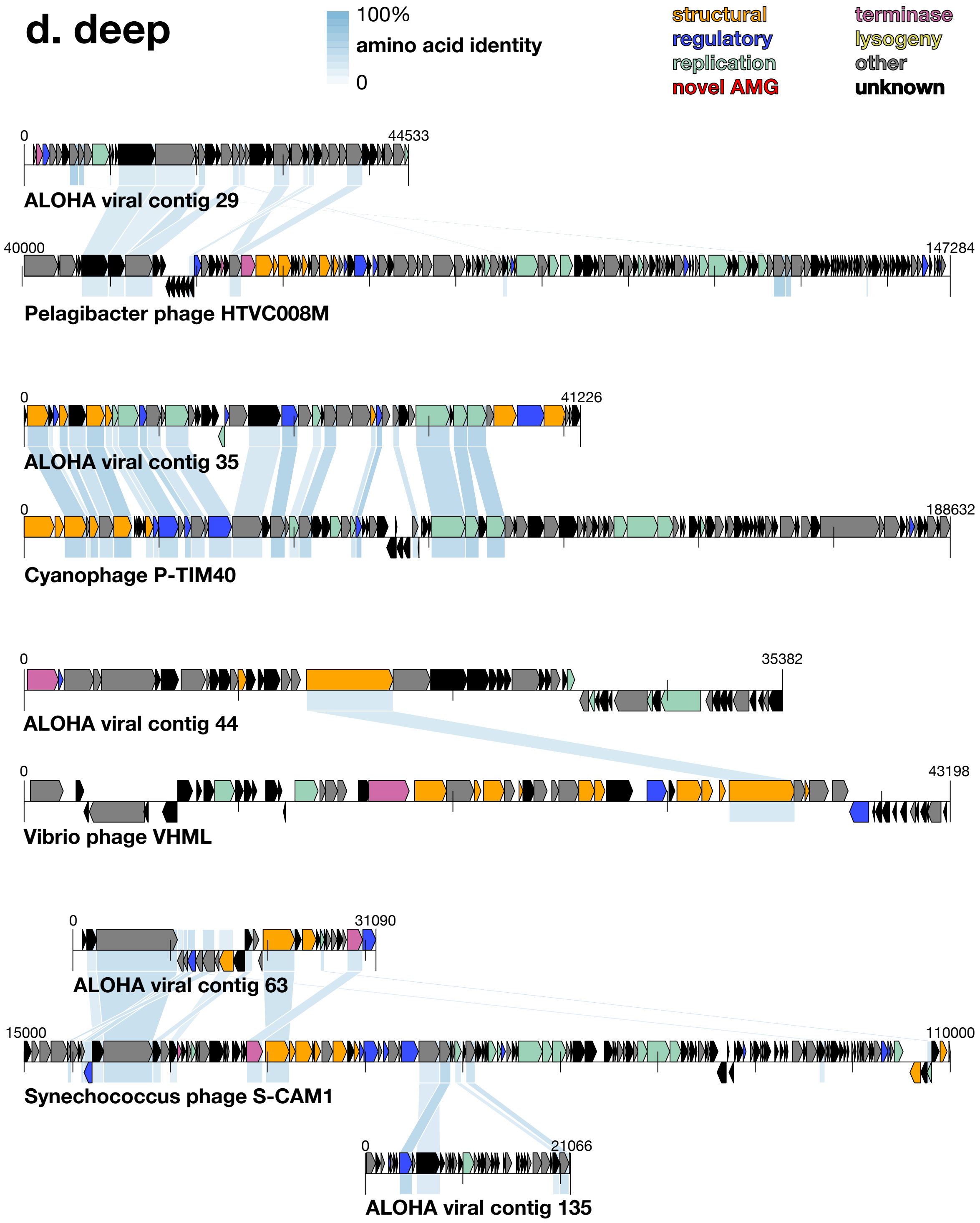
d. deep
100%
amino acid identity
0
structural
regulatory
replication
novel AMG
terminase
lysogeny
other
unknown
0
44533
ALOHA viral contig 29
40000
147284
Pelagibacter phage HTVC008M
0
41226
ALOHA viral contig 35
0
188632
Cyanophage P-TIM40
0
35382
ALOHA viral contig 44
0
43198
Vibrio phage VHML
0
31090
ALOHA viral contig 63
15000
110000
Synechococcus phage S-CAM1
0
21066
ALOHA viral contig 135

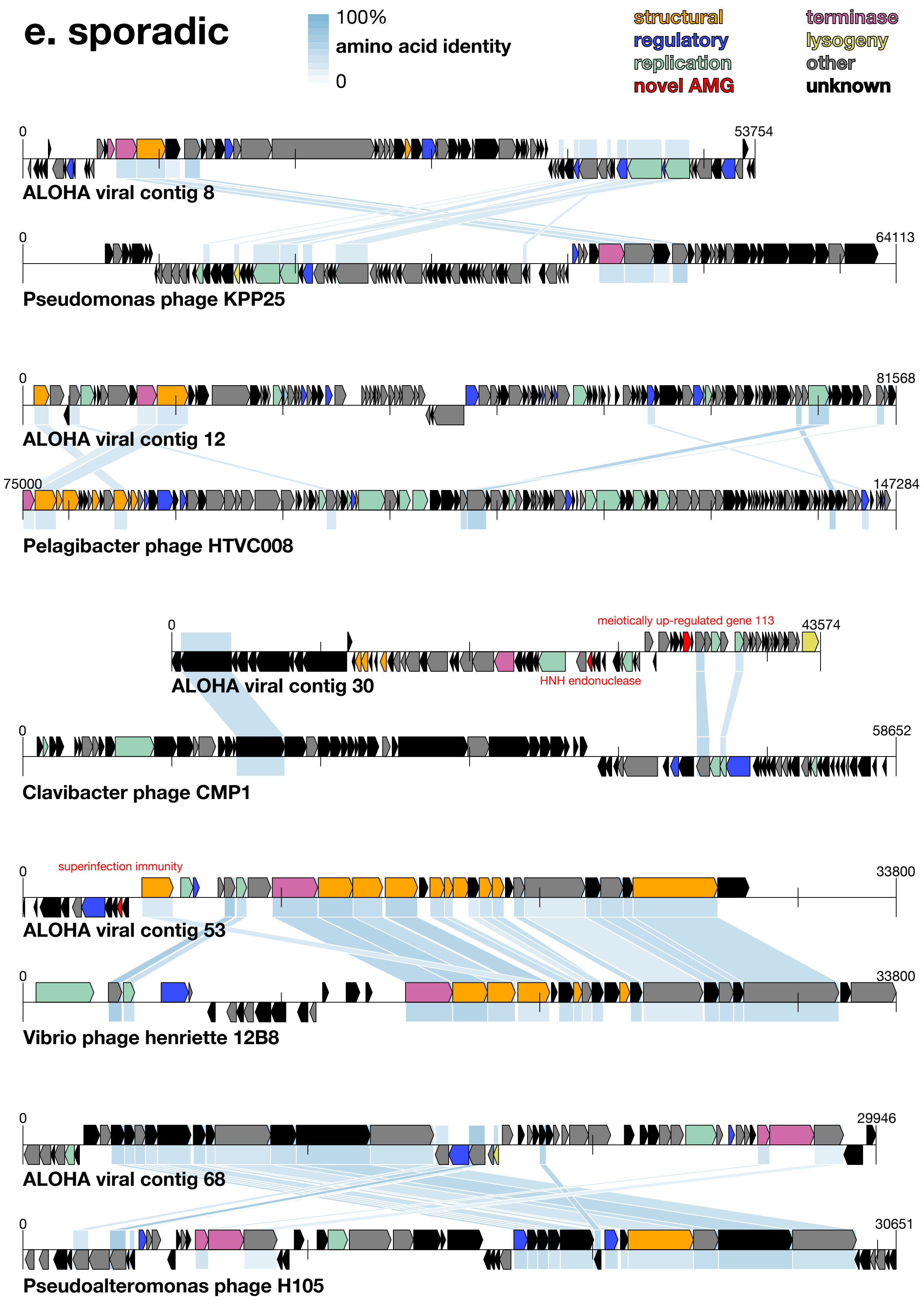

e. sporadic
100%
amino acid identity
0
structural
regulatory
replication
novel AMG
terminase
lysogeny
other
unknown
0
53754
ALOHA viral contig 8
0
64113
Pseudomonas phage KPP25
0
81568
ALOHA viral contig 12
75000
147284
Pelagibacter phage HTVC008
0
meiotically up-regulated gene 113
43574
ALOHA viral contig 30
HNH endonuclease
0
58652
Clavibacter phage CMP1
0
superinfection immunity
33800
ALOHA viral contig 53
0
33800
Vibrio phage henriette 12B8
0
29946
ALOHA viral contig 68
0
30651
Pseudoalteromonas phage H105
