# Supplementary material for: Bacteriophage Distributions and Temporal Variability in the Ocean’s Interior
Source: mBio. 2017 Nov 28;8(6):e01903-17. doi: 10.1128/mBio.01903-17 (PMC5705922; doi:10.1128/mBio.01903-17)
Supplement: TABLE S3 [file mbo006173616st3.pdf]

Supplementary Table 3. List of 37 novel genes in marine phage identified from 625 curated contigs co-located with viral structural genes (PFAM bit score >10) that has not been reported in other previously sequenced viromes (13, 14). 19 putative novel auxiliary metabolic genes are noted with an asterisk (*).

PFAM	annotation
PF00147*	Fibrinogen beta and gamma chains, C-terminal globular domain
PF00782*	Dual specificity phosphatase, catalytic domain
PF01206*	Sulfurtransferase TusA
PF01658*	Myo-inositol-1-phosphate synthase
PF01758*	Sodium Bile acid symporter family
PF01812*	5-formyltetrahydrofolate cyclo-ligase family
PF01918	Alba
PF02597*	ThiS family
PF03602*	Conserved hypothetical protein 95
PF04892*	VanZ like family
PF05262*	Borrelia P83/100 protein
PF06855*	YozE SAM-like fold
PF07902	gp58-like protein
PF08279	HTH domain
PF09382	RQC domain
PF12385*	Papain-like cysteine protease AvrRpt2
PF13022	Helix-turn-helix of insertion element transposase
PF13231*	Dolichyl-phosphate-mannose-protein mannosyltransferase
PF13455	Meiotically up-regulated gene 113
PF14279	HNH endonuclease
PF14326	Domain of unknown function (DUF4384)
PF14373*	Superinfection immunity protein
PF15943*	Putative antitoxin of bacterial toxin-antitoxin system, YdaS/YdaT
PF16075	Domain of unknown function (DUF4815)
PF16190*	Ubiquitin-activating enzyme E1 FCCH domain
PF16243	Sm_like domain
PF16363*	GDP-mannose 4,6 dehydratase
PF16510	Phage P22-like portal protein
PF16724	T4-like virus Myoviridae tail sheath stabiliser
PF16778	Phage tail assembly chaperone protein
PF16786	Recombination enhancement, RecA-dependent nuclease
PF16790	Bacteriophage clamp loader A subunit
PF16805	Phage late-transcription coactivator
PF16861*	Carbamoyltransferase C-terminus
PF16868*	NMT1-like family
PF16945	Putative lactococcus lactis phage r1t holin
PF17212	Tail tubular protein
