# Supplementary material for: Bacteriophage Distributions and Temporal Variability in the Ocean’s Interior
Source: mBio. 2017 Nov 28;8(6):e01903-17. doi: 10.1128/mBio.01903-17 (PMC5705922; doi:10.1128/mBio.01903-17)
Supplement: TABLE S4 [file mbo006173616st4.pdf]

Supplementary Table 4. List of 4 persistent ALOHA viral contigs with hits to surface phages in a 2015 dataset near Station ALOHA (24) with more than half of its genes hitting an average amino acid identity of >60%.

<table>
<tr><th>contig</th><th>reference</th><th>proportion gene hits</th><th>AAI %</th></tr>
<tr><td>AVC003</td><td>VS12|size37975</td><td>0.739130434783</td><td>68.9752941176</td></tr>
<tr><td>AVC011</td><td>VS13|size35890</td><td>0.674418604651</td><td>75.8634482759</td></tr>
<tr><td>AVC017</td><td>VS13|size35890</td><td>0.608695652174</td><td>66.0454761905</td></tr>
<tr><td>AVC063</td><td>VS7|size43665</td><td>0.888888888889</td><td>62.0079166667</td></tr>
</table>
