# Supplementary material for: Bacteriophage Distributions and Temporal Variability in the Ocean’s Interior
Source: mBio. 2017 Nov 28;8(6):e01903-17. doi: 10.1128/mBio.01903-17 (PMC5705922; doi:10.1128/mBio.01903-17)

Supplementary Figure 4. Depth profile of phage marker proteins identified in the Station ALOHA non-redundant gene catalogue (domain bit score >50) using hidden markov models generated with manually curated set of proteins from NCBI. Each circle represents a sample mean and each vertical bar represents a depth mean. Depth profile of prophage markers are shown in closed circles: a) integrase, b) CI repressor, and c) excisionase. Depth profile of phage markers are shown in open circles: d) DNA polymerase e) terminase f) capsid g) tail fiber. The copy number of marker genes per cell genome equivalent is calculated using marker gene coverage normalized to average coverage of 10 single-copy bacterial marker genes.

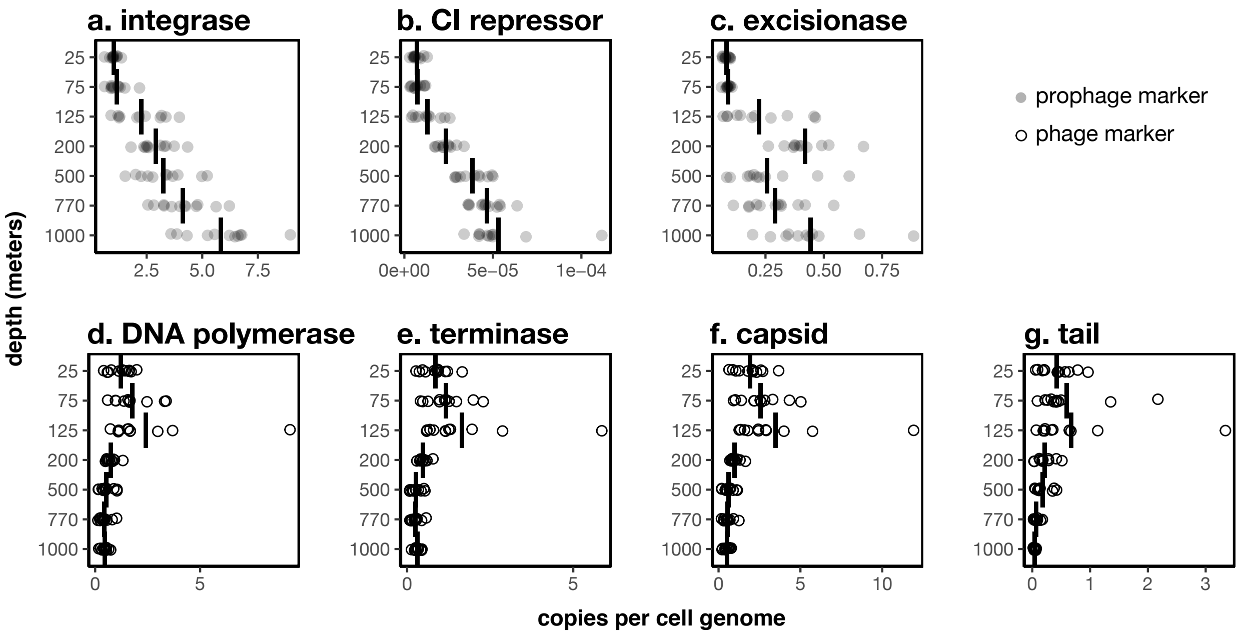

Supplement: FIG S4 [file mbo006173616sf4.pdf]
