# Supplementary material for: Bacteriophage Distributions and Temporal Variability in the Ocean’s Interior
Source: mBio. 2017 Nov 28;8(6):e01903-17. doi: 10.1128/mBio.01903-17 (PMC5705922; doi:10.1128/mBio.01903-17)

Supplementary Figure 5. Bubble plot of proportion of ALOHA gene catalogue genes hitting to four groups of a) known phage and b) associated bacteria. Hits assigned based on protein-protein LAST against RefSeq75 database at >60% amino acid identity. Proportion of genes is normalized by gene coverage and is scaled by area.

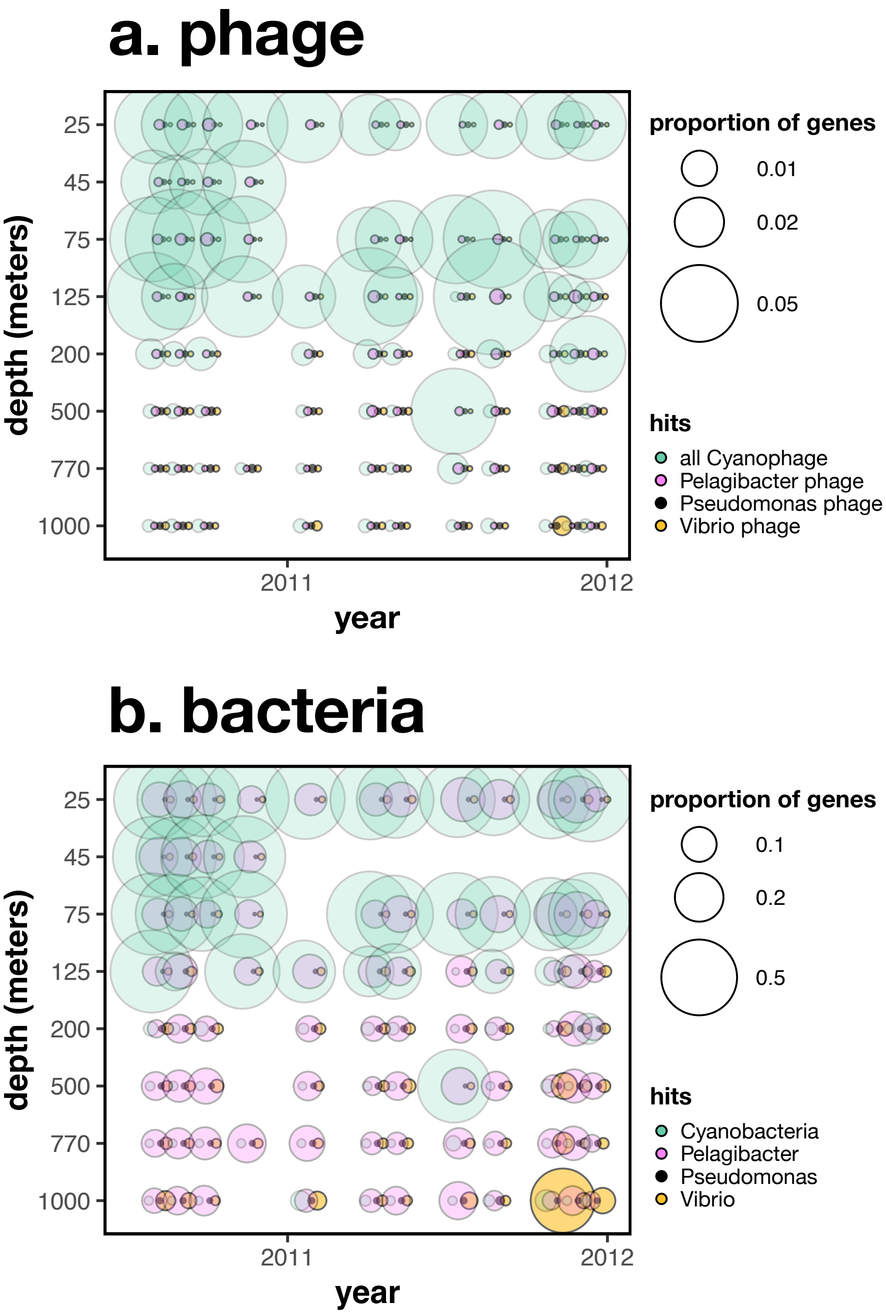

Supplement: FIG S5 [file mbo006173616sf5.pdf]
